# Transcriptomic profiling of human skin biopsies in the clinical trial setting: A protocol for high quality RNA extraction from skin tumours

**DOI:** 10.12688/wellcomeopenres.14360.1

**Published:** 2018-04-23

**Authors:** Marina Danilenko, Robert Stones, Neil Rajan

**Affiliations:** 1 Institute of Genetic Medicine, University of Newcastle upon Tyne, Newcastle upon Tyne, NE1 3BZ, UK; 2 Department of Dermatology, Royal Victoria Infirmary, Newcastle upon Tyne, NE1 4LP, UK

**Keywords:** Skin biopsy, tumour, CYLD, RNA sequencing, cancer, clinical trial, transcriptomics.

## Abstract

Transcriptomic profiling of skin disease using next generation sequencing allows for detailed information on aspects of RNA biology including gene expression, non-coding regulatory elements and gene splicing. The application of RNA sequencing to human skin disease and cancer is often hampered by degraded RNA. Here we describe a protocol that allows for consistently intact RNA to be extracted from snap frozen skin biopsy samples, which has been validated in a clinical trial setting.

Human skin tumour punch biopsies (n=28) ranging from 4-6mm in diameter were obtained from 14 patients with an inherited skin tumour syndrome (CYLD cutaneous syndrome) and frozen in liquid nitrogen prior to being stored at -80°C. These samples were then subject to cyrostat sectioning, allowing for histological assessment, and were homogenised using a bead-based lysis platform. RNA extraction was performed using a silica column-based system. RNA concentration was measured using fluorescent quantitation and RNA integrity assessed using microfluidic gel electrophoresis. We also processed normal skin biopsies using the same protocol (n=10). The mean RNA integrity score of the tumour and normal samples was 9.5, and the quantity of RNA obtained from the small amounts of tissue used exceeded requirements for RNA-seq library generation.

We propose that the method of RNA extraction suggested here allows for transcriptomic profiling from small pieces of human tissue without the need for PCR amplification during library preparation. This protocol could be utilised in healthy and diseased skin to improve mechanistic understanding in a range of human skin diseases.

## Introduction

Skin has evolved as a protective barrier against environmental stresses such as physical damage, ultraviolet radiation exposure and pathogenic infections. It is a complex tissue, consisting of many cell types, including keratinocytes, immune cells, melanocytes, fibroblasts, adipocytes, nerve fibres, smooth muscle and endothelial cells ( Cole *et al.*, 2001). Skin diseases may involve any of these constituent cells, and transcriptomic profiling of diseased human skin can inform understanding of disease pathogenesis. The accessible nature of the skin, unlike tissues such as the brain or liver, and the low patient impact of skin biopsy, make the use of transcriptomics in human skin particularly attractive. Whole punch biopsies have been studied using transcriptome-wide approaches, giving insights into the inflammatory skin disease psoriasis ( Zolotarenko *et al.*, 2016).

Reliably obtaining high quality RNA of sufficient quantity from small samples typical of those derived from punch biopsies is, however, problematic. The reasons for this are diverse and include suboptimal tissue disruption and endogenous RNase activity, resulting in skin being a tissue that is amongst the most challenging to obtain intact RNA from ( Walker *et al.*, 2016)( Kaufmann *et al.*, 1980). RNA is also a highly unstable molecule. Once extracted from cells or tissues it has a very short half-life and is easily degraded ( Brooks, 1998; Tan & Yiap, 2009). RNA is chemically unstable due to the presence of a hydroxyl group at the 2’ and thus is highly susceptible to hydrolysis by ribonuclease enzymes. The hydrolysis or cleavage of RNA can also occur spontaneously, without the presence of a catalyst or enzyme ( Elliott & Ladomery, 2011). Together, these challenges may discourage the inclusion of transcriptomics in clinical trials involving human skin, where such information may offer mechanistic insights.

Methods of RNA extraction from human skin have been evolving. Early approaches required large amounts of tissue, and were not amenable to medium/high throughput lysis, as each sample had to be disrupted in liquid nitrogen ( Hipfel *et al.*, 1998; Kaufmann *et al.*, 1980). Whole punch biopsies of the skin have been successfully disrupted by low throughput devices, such as the Kinematica Polytron 1300D homogenizer and FastPrep120 bead beater ( Berglund *et al.*, 2007). 4 mm skin biopsies have also been subjected to ammonium thiocyanate-induced dermo-epidermal separation and subsequently homogenized using bead-based lysis ( Clemmensen *et al.*, 2009); however the RNA integrity may have been suboptimal due to tissue processing. Importantly none of these approaches allow for histological assessment of the skin sample, which is relevant in interpreting sequencing results of diseased skin. Laser capture microdissection is an alternative method with histological information, with the limitations being cost of laser capture microscopy equipment, small amount of RNA obtained, and RINs that may be lower (circa 7.0) ( Butler *et al.*, 2016). An additional amplification stage is also often required when starting from small amounts of RNA during library generation, which may introduce an element of bias ( Jackson *et al.*, 2014).

Here we report an efficient method of RNA extraction from skin tumours obtained using punch biopsies under local anaesthetic in a clinical trial involving patients with an inherited skin tumour syndrome. We also validated this method in normal human skin biopsies. Downstream analysis demonstrated high yield and RNA integrity, allowing for transcriptomic profiling using RNA sequencing.

## Methods

### Ethics

Regulatory approvals for the present study were sought and obtained from an ethics review committee (National Research Ethics Service Committee North East-Tyne and Wear Ref:14/NE//080 and 06/1059) and the Medicines Health Regulatory authority (EudraCT: 2014-001342-21). Patients provided written, informed consent for biopsy and use of their tissue samples for research. This study used samples collected as part of a clinical trial, registered at ISCRTN 75715723. The study was performed in accordance with the Declaration of Helsinki.

### Sample collection

Tumours scheduled for biopsy as part of the trial protocol were identified using tumour maps and photographs. Tumour identifiers were labelled onto cryovials in advance of the procedure. Skin biopsies were performed under local anaesthetic, and one sample was taken at a time. The biopsy was transferred to a labelled cryovial, with identifiers checked together with a research nurse. The sample was then immediately immersed in liquid nitrogen. All samples were logged in accordance with standard operating procedures and stored in a -80°C freezer within a designated Human Tissue Authority freezer that is subject to temperature monitoring.

### Sample processing

**Figure 1.**
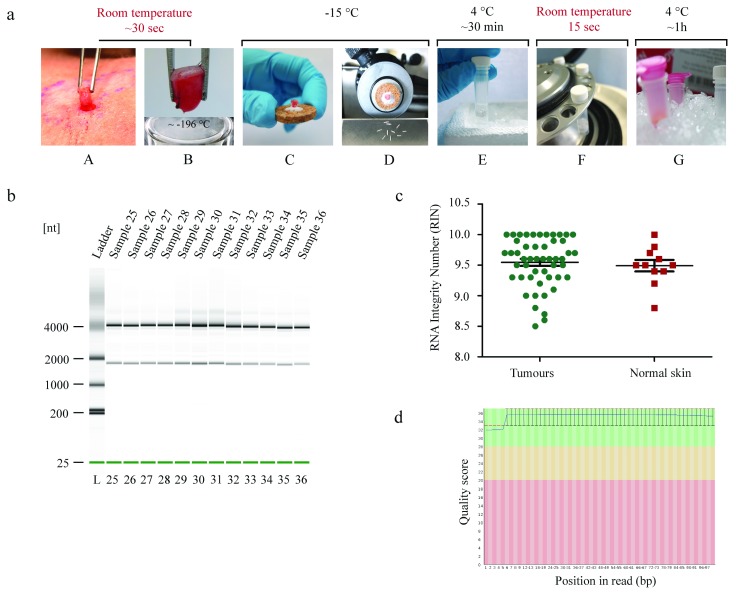
A method for extracting high quality RNA from small skin biopsy samples which is suitable for RNA sequencing. ( **a**) Diagram indicating key steps in the work flow, and the need to keep samples cold throughout. (A) Human skin punch biopsy (B) Freezing of sample within 30 seconds. (C–D) Cryostat sectioning of punch biopsy and curls obtained. (E–F) Addition of lysis buffer to single use bead tube, followed by homogenisation in the bead-based lysis machine where up to 24 samples can be processed at once. (G) Silica spin column based RNA extraction performed at 4 degrees. ( **b**) Microfluidic gel analysis of total RNA demonstrates distinct 18S and 28S ribosomal bands, consistent with the high RNA integrity scores ( **c**) demonstrated across samples. ( **d**) FASTQc assessment of reads indicate high quality reads in libraries developed from this dataset.

A full working protocol is detailed in Supplementary File 1, and the key steps are outlined here. Standard precautions to prevent contamination with RNAses were employed. The sample was removed from the freezer and transferred on dry ice to the cryostat. The sample identifier was used to label slides and preweighed lysis tubes, which were supplied prefilled with beads (Precellys). Skin punch biopsy samples of tumours (4–6 mm diameter) were mounted on a cork piece using cutting compound (OCT), and then serially sectioned ( Figure 1). Each skin biopsy was cryosectioned at two levels and ten 30-micron curls were taken from each level for RNA extraction; two were taken from each biopsy. Sections were then taken for histology and applied to a standard slide, and subject to haematoxylin and eosin staining. Following cryosectioning, material was immediately placed into the bead lysis tube, weighed again and then cold lysis buffer (RLT) containing beta-mecaptoethanol was added (Qiagen RNeasy Micro Kit No 74004). The tube was frozen on dry ice and then returned to the -80°C freezer until RNA extraction was performed.

### RNA extraction and quality control

The protocol supplied with the silica spin column kit (Qiagen RNeasy Micro Kit No 74004, Qiagen UK) was followed with modifications, as indicated in the working protocol ( Supplementary File 1). Briefly, bead tubes were taken from the -80°C freezer on dry ice. Tissue was then homogenized in a Precellys Evolution homogeniser (Bertin, France) for 20 seconds at 5500 bpm. After homogenization, the lysate was applied to the column, and then wash steps were performed. On-column DNase digestion was performed for 7 minutes at room temperature. Further washes were performed before RNA was eluted from the column with RNase-free water. All protocol steps were performed in a cold centrifuge at 4°C at 15000 rpm apart from the DNase incubation step stated above. Eluted RNA was measured using the Qubit BR assay kit (Thermofisher, UK). RNA quality was measured using a microfluidic gel electrophoresis chip (Bioanalyer RNA 6000 Nano Chip, Agilent UK). RNA integrity numbers were obtained with the software provided (2100 Expert Software: Revision B.02.09 (SR1)) with the Agilent 2100 Bioanalyser (Agilent, UK) ( Schroeder *et al.*, 2006).

### Transcriptomic analyses

To obtain the cytokeratin signatures of differentially expressed genes in cylindroma and spiradenoma compared to control skin, three tumour transcript files were compared to three control skin sample files. RNA from each sample was used to generate sequencing libraries using the Illumina Truseq stranded mRNA kit. Libraries were sequenced using an Illumina Hiseq 2500, giving 45 million paired end reads per sample which were 100 bp in length. FASTQ files were checked for quality using FASTQc and aligned using the splice aware aligner program STAR (v. 2.5.2b) to generate alignment files ( Dobin *et al.*, 2013). The read counts for each sample file were obtained using the R package Subread (v.1.28.1) ( Liao *et al.*, 2013). Differential gene expression analysis was carried out using DeSeq2 v.1.18.1 ( Love *et al.*, 2014). Cytokeratin genes that were differentially expressed with a false discovery rates of <0.05 after correction for multiple hypothesis testing are listed in Table 3.

## Results

### Consistent high-quality RNA is extracted from multiple skin tumours

28 skin biopsies were cryosectioned and material with an average weight of 10.4mg (range 5 –14 mg) was obtained at two levels in each biopsy Figure 1a. RNA was obtained from a total of 56 levels, with total yield exceeding the requirements for library preparation for RNA sequencing (yields typically >500ng) in the majority of samples. Sections taken for histology were stained using haematoxylin and eosin, and confirmed adjacent curls were taken from cylindroma and spiradenoma in 25 out of 28 of biopsies; three biopsies demonstrated trichoepithelioma. In 3 out of 56 levels, RNA was not obtained and this correlated with histology reflecting relatively acellular dermis beneath the level of the tumour. The range of concentrations and quality of RNA extracted from tumours are indicated in Table 1 and Figure 1b and c, indicating a mean RIN of 9.5 (range 8.5–10). Control skin sample yields and integrity (mean RIN 9.5; range 8.8–10) are indicated in Table 2.

**Table 1.** RNA concentrations and integrity in the 28 skin tumour biopsies studied, with 2 samples taken per biopsy. Qualitative and quantitative measurements of the total RNA isolated from normal skin punch biopsies.

Tumour	Samples	Concentration (ng/ul)	RNA integrity
1	1	129.6	9.7
	2	17.2	-
2	3	63.2	9.3
	4	too low/undetectable	-
3	5	326	9.3
	6	208	9.3
4	7	118	9.3
	8	76	9
5	9	208	10
	10	142	9.8
6	11	115.6	9.9
	12	29.2	8.5
7	13	272	10
	14	56	10
8	15	182.8	9.6
	16	169.2	9.6
9	17	147.4	9.5
	18	16.6	-
10	19	99.6	9.4
	20	105.6	9.3
11	21	98	9.5
	22	12.6	-
12	23	41.4	9.1
	24	82.8	9.7
13	25	282	10
	26	228	9.9
14	27	308	9.9
	28	304	10
15	29	258	10
	30	370	10
16	31	800	10
	32	1120	10
17	33	197.8	9.7
	34	244	9.6
18	35	286	9.6
	36	148	9.5
19	37	234	9.4
	38	250	9.6
20	39	362	9.7
	40	254	9.6
21	41	252	10
	42	55	10
22	43	49.6	9.8
	44	26	9.5
23	45	17.2	9.2
	46	21	9.8
24	47	25	8.6
	48	too low/undetectable	-
25	49	360.8	9.7
	50	194.8	9.6
26	51	110.6	9.3
	52	50.6	9
27	53	15.8	8.7
	54	16.6	9
28	55	26.6	8.8
	56	too low/undetectable	-
**Average:**		**180.8**	**9.5**

**Table 2.** RNA concentrations and integrity of 10 normal skin samples studied. Qualitative and quantitative measurements of the total RNA isolated from normal skin punch biopsies.

Samples	Concentration (ng/ul)	RNA Integrity
1	25.2	9.6
2	17	8.8
3	18.6	9.2
4	23	10
5	38.6	9.5
6	20.2	9.5
7	32	9.4
8	36.6	9.4
9	40	9.8
10	21.8	9.7
**Average:**	**27.3**	**9.5**

**Table 3.** Expression of known cytokeratin signatures of differentially expressed genes in cylindroma and spiradenoma compared to control skin.

Gene	log2 Fold Change	Prob. FDR	Description
*KRT13*	3.94	1.37E-11	Keratin 13
*KRT17P2*	2.44	0.011702576	Keratin 17 pseudogene 2
*KRTCAP3*	1.05	0.012251062	Keratinocyte associated protein 3
*TCHP*	-0.83	0.022666568	Trichoplein keratin filament binding
*KRT15*	-0.89	0.031124672	Keratin 15
*KRT3*	-1.56	0.016500418	Keratin 3
*KRT9*	-1.71	0.002468716	Keratin 9
*KRT5*	-1.74	0.000610354	Keratin 5
*KRT8P26*	-1.96	0.012588263	Keratin 8 pseudogene 26
*KRT80*	-1.96	0.017781638	Keratin 80
*KRT19*	-2.07	0.035571416	Keratin 19
*KPRP*	-2.20	0.013678232	Keratinocyte proline rich protein
*KRTDAP*	-2.35	0.001196344	Keratinocyte differentiation associated protein
*KRT78*	-2.39	0.00676354	Keratin 78
*KRT10*	-2.46	0.00126146	Keratin 10
*KRT14*	-2.48	0.00000538	Keratin 14
*KRT1*	-2.55	0.000588345	Keratin 1
*KRT77*	-2.67	0.00000744	Keratin 77
*KRT72*	-2.71	0.000336864	Keratin 72
*KRT27*	-2.76	0.005439881	Keratin 27
*KRT73*	-3.37	3.44E-08	Keratin 73
*KRT31*	-3.89	4.65E-11	Keratin 31
*KRT2*	-4.02	0.000000475	Keratin 2

### RNA sequencing validates tumour transcriptomic signature

Reads derived from next generation sequencing were subject to quality control using FASTQc ( Figure 1d), and this demonstrated high quality scores, consistent with the high integrity RNA used. Differential gene expression studies focussed on known differentially expressed signature cytokeratin genes in cylindroma and spiradenoma, skin tumours seen in CYLD cutaneous syndrome ( Brown *et al.*, 2018). This confirmed that the recognised cytokeratin signature was expressed in these tumour transcriptomes ( Table 3).

## Discussion

Due to its high sensitivity and resolution, RNA sequencing is a powerful tool for investigation of skin disease. It can provide mechanistic insights behind disease pathogenesis, which may offer prognostic information, prediction of response to treatments and the potential for developing novel therapies ( Gilmore, 2013). To overcome the inherent difficulties of obtaining high integrity RNA, there are in general, four crucial steps required during extraction: cells or tissues should be completely homogenised; nucleoprotein complexes should be disrupted; RNase should be inactivated; contamination including carbohydrate, lipids, protein and other nucleic acid should be avoided ( Buckingham & Flaws, 2007; Doyle & Doyle, 1990).

We describe a novel method, incorporating cryostat sectioning, resulting in partial disruption of the tissue, whilst simultaneously allowing us to take 8 μm sections for histological assessment. We used bead-based tubes that allowed for up to 24 samples to be processed simultaneously. Importantly, we were able to work with small amounts of skin, which were robustly disrupted using the bead-based lysis system we employed. This prevented carryover of incompletely homogenised material and obstruction of spin columns used for RNA extraction. The bead-based lysis system we employed had a range of disruption settings and after optimisation with a range of bead sizes, we chose a medium bead mix (CK28 Mix), and lysis of 20 seconds at 5500 rpm in lysis buffer as this gave optimal RNA extraction and RINs.

After skin homogenisation, there are several established methods for RNA extraction. Phenol/chloroform extraction, one of the commonly used techniques, has potential for phenol contamination of the samples, which is often reported ( Oñate-Sánchez & Vicente-Carbajosa, 2008). Therefore, we employed a silica column-based RNA extraction methodology ( Sellin Jeffries *et al.*, 2014). This approach suited our protocol, which required small volumes of lysis buffer, and elution of RNA performed in 14ul of water. This typically resulted in highly concentrated samples and thus satisfied the requirements of most RNA library preparation kits for next generation sequencing.

Given RNA’s sensitivity to temperature, we developed a protocol where the skin sample is kept as cold as possible throughout. The tumour samples were first snap frozen in liquid nitrogen, and then cryosectioned at a temperature of -20°C. During rapid homogenisation in our protocol, the tumour material was exposed to ambient temperatures for only a few minutes. This was immediately followed by RNA extraction in a precooled centrifuge at 4°C. Spin columns were incubated on wet ice between centrifugation steps and eluted RNA was immediately frozen after RNA extraction was complete.

In summary, we developed a method for efficient extraction of RNA from small cryosectioned pieces of human skin with histological data from adjacent tissue sections. We validated this method in human skin tumours samples in a clinical trial setting. This protocol could be utilised in healthy and diseased skin to improve mechanistic understanding in a range of human skin disease and cancer.

## Data availability

FASTQ files (controls [n=3] and tumours [n=3] used to generate validation signatures), sample description files and uncropped gel images are available at Open Science Framework: http://doi.org/10.17605/OSF.IO/5YX96 ( Rajan, 2018).

Data are available under the terms of the Creative Commons Zero "No rights reserved" data waiver (CC0 1.0 Public domain dedication).

## Supplementary material

Supplementary File 1: Working protocol.
