# Supplementary material for: Transcriptomic profiling of human skin biopsies in the clinical trial setting: A protocol for high quality RNA extraction from skin tumours
Source: Wellcome Open Res. 2018 Apr 23;3:45. [Version 1] doi: 10.12688/wellcomeopenres.14360.1 (PMC5989147; doi:10.12688/wellcomeopenres.14360.1)
Supplement: Supplementary file 1 [file wellcomeopenres-3-15623-s0000.tgz › 26627eab-a18c-4a61-a9b2-a712b66c3424.docx]

**Transcriptomic profiling of human skin biopsies in the clinical trial setting: A protocol for high quality RNA extraction from skin tumours**

**Do the following once when the Qiagen kit is first opened.**

- **The whole kit should be stored at 4°C and taken out prior to experiment so that reagents are cold.**
- Add **4 volumes of ethanol (96-100%) to buffer RPE** for a working solution.
- Prepare the **DNase I stock**: dissolve the lyophilized **DNase I in 550ul RNase-free water**. Open/close the bottle with DNAse carefully, not touching the lid. Mix gently by inverting; do not vortex. As stock cannot be frozen and defrosted many times, aliquot the stock to PCR tubes, 10ul into each and freeze. Store at -20°C for up to 9 months.

**Do the following before each extraction:**

- Add **10ul of β-ME** (in the fume hood) per **1ml of Buffer RLT** in fume hood. (350ul of buffer is used for tissue with weight less than 2mg, 600ul for higher weight). **Once prepared, RLT should be placed on wet ice or kept at 4°C**
- 70% ethanol should be prepared fresh for each extraction. 70% ethanol should be made with DEPC treated H2O. A separate ethanol bottle should be dedicated for RNA work only. As other reagents, 70% ethanol should be kept on wet ice after being made.
- Prepare the centrifuge to start the experiment with 4°C.
- Prelabel Precellys tubes and weigh on a fine balance.

**During cryosectioning:**

- The brushes/forceps/bench/gloves have to be cleaned with ethanol and RNAse ZAP before each new tumour.
- Precellys tubes should be placed onto dry ice prior cutting. Each tube should be taken out only prior to putting the curls.
- Take 10x30um slices for each sample at each level, and an 8um slice for histology.
- Return Precellys tubes to dry ice then weigh again.
- RLT+ β-ME should be kept on wet ice. It should be added to each tube after weighing. Once it is added, tubes should be kept on wet ice.

**Precellys:**

- Lysis should be done immediately after cutting.
- 1x20 seconds at 5500 on Precellys Evolution, then return to wet ice.
- Commence RNA extraction

**RNA Extraction with RNeasy Micro Kit:**

- General notes
  - All steps should be performed in a cold centrifuge.
  - Between all steps samples should be on wet ice.
  - Take each individual tube out, add the solution, place it back on ice.
  - DNAse incubation step is the only one performed in a room temperature.
  - Keep all the reagents added next cold so that to reduce any warming as much as possible.

**Steps:**

1. Add **1 volume (e.g. 350ul; 600ul if higher weight) of 70% ethanol**. Mix well by pipetting.
2. Transfer the sample (with any precipitate) to an **RNeasy MinElute spin column placed into a 2ml collection tube** (supplied). Centrifuge for 1 min at 15000xg (or full speed). Discard the flow.
3. Add **350ul Buffer RW1**. Centrifuge for 1-2 min at 15000xg (or full speed). Discard the flow through.
4. Add **10ul DNase I stock solution** to 70ul buffer RDD. Mix by inverting the tube. Add the mix (80ul) to the column. Keep on the bench at ambient temperature for 7-8 minutes. Add 350ul RW1 buffer to the column. Centrifuge for 1 min at 15000xg (or full speed). **Discard the collection tube.**
5. Place the column into a **new 2ml collection tube**. Add **500ul Buffer RPE** to the column. Centrifuge for 1 min at 15000xg (or full speed). Discard the flow.
6. Add **500ul of 80% ethanol** to the column. Centrifuge for 2 min at 15000xg (or full speed). **Discard the collection tube.**
7. Place the column to a **new 2ml collection tube** and centrifuge for 5 min with **open lid** to dry the membrane. **Discard the collection tube.**
8. Place the column in **a new 1.5ml collection tube** (supplied). Add **14ul RNase–free water** directly to the center of the spin column membrane. Wait for 1 minute. Close the lid gently and centrifuge for 1 minute at full speed to elute the RNA.
9. Quantify RNA using Qubit BR assay.
10. Put the samples onto dry ice as soon as possible, and store at -80°C.

**Acknowledgements**

This work was supported by the Wellcome Trust [WT097163MA]; Wellcome Trust and Department of Health under the Health Innovation Challenge Fund [100935/Z/13/Z].

***Disclaimer****: This publication presents independent research commissioned by the Health Innovation Challenge Fund (HICF-R7-395), a parallel funding partnership between the Wellcome Trust and the Department of Health. The views expressed in this publication are those of the author(s) and not necessarily those of the Wellcome Trust or the Department of Health. The protocol is published to improve the quality of research based on human skin transcriptomics. It is not designed for clinical diagnostic or therapeutic use and is for research applications only. Please ensure all local and national regulatory requirements relating to the relevant use of this protocol are satisfied.*
