# Supplementary material for: Transcriptomic profiling of human skin biopsies in the clinical trial setting: A protocol for high quality RNA extraction from skin tumours
Source: Wellcome Open Res. 2018 Apr 23;3:45. [Version 1] doi: 10.12688/wellcomeopenres.14360.1 (PMC5989147; doi:10.12688/wellcomeopenres.14360.1)
Supplement: Peer review history

# Peer review: Referee response for version 1

**DOI:** 10.21956/wellcomeopenres.15623.r32973

**Published:** 2018-06-20

**Authors:** Tarl W. Prow

**Affiliations:** 1 Future Industries Institute, University of South Australia, Adelaide, SA, Australia

The article describes a practical and highly effective means to isolate RNA from fresh clinical skin samples for downstream transcriptome analysis.

 The major issue that I see with the manuscript is the lack of text describing previous studies using the conventional approach to RNA isolation. In my view, this manuscript describes a methodology to generate extremely high quality RNA isolates. Please describe the RIN from other methods in the published literature so that this can be appreciated. The other major point that I see is that the usual work-flow in dermatology is to paraffin embed samples and then isolate RNA or DNA from sections. The authors should comment on the RNA quality and sequencing considerations from this approach.

 Minor note: It may be worth mentioning that RPKM based assessments of mRNA abundance have issues with inconsistency and that TPM may be the better model. This is only a minor issue.

 In summary, this is a well written manuscript that has broad application and immediate relevance. All of my comments can be addressed within the document. No further experiments are needed in my opinion.

I have read this submission. I believe that I have an appropriate level of expertise to confirm that it is of an acceptable scientific standard.

# Peer review: Referee response for version 1

**DOI:** 10.21956/wellcomeopenres.15623.r33203

**Published:** 2018-06-01

**Authors:** Verena Wally, Mo Wimmer

**Affiliations:** 1 EB House Austria, Research Program for Molecular Therapy of Genodermatoses, Department of Dermatology, University Hospital Salzburg, Paracelsus Medical University, Salzburg, Austria; 2 EB House Austria, Research Program for Molecular Therapy of Genodermatoses, Department of Dermatology, University Hospital Salzburg, Paracelsus Medical University, Salzburg, Austria

The paper "Transcriptomic profiling of human skin biopsies in the clinical trial setting: A protocol for high quality RNA extraction from skin tumours" talks about a novel method to isolate high quality RNA from skin biopsies in a clinical trial setting.

 The method is well described and provides information on several aspects to consider in the course of respective sample preparation.

 However, we would like to address some points we noticed:

1. Authors state that this is a new method, circumventing several critical aspects of RNA extraction. However, we feel that the novelty is not well enough discussed. A comparison of the key aspects with some aspects of other standard procedures would facilitate a better understanding of the advantages. Optimally, simultaneous RNA preparation with a commonly used method would have been interesting as a methodical control.
2.  The problem statement should be elaborated more in detail in the introduction.
3. A statement on why these skin tumours were chosen for the establishment of the method should be included and how patients could benefit from this method in the clinical trial setting.

 

 Minor points:

1. Some more information on the gene expression signature should be given in the text and what relevance the validation of the expression profile has in a clinical trial setting.
2. Table 3: A short statement on the results shown in the table would be helpful in the table description.
3. Figure 1D is not comprehensively described.
4. Why is the final average RNA concentration 10-fold higher in tumour samples compared to normal skin biopsies? (Tables 1 and 2)
5. Table 1. Subtitle: … is “normal” skin biopsies correct?
6. Some typos / spaces in the paper. (eg. abstract paragraph 2: c **YR**ostat; page 5: **A** false discovery rate **S**, …)

We have read this submission. We believe that we have an appropriate level of expertise to confirm that it is of an acceptable scientific standard.

# Peer review: Referee response for version 1

**DOI:** 10.21956/wellcomeopenres.15623.r33121

**Published:** 2018-05-24

**Authors:** Irene M. Leigh

**Affiliations:** 1 Centre for Cutaneous Research, Barts and the London School of Medicine and Dentistry, Queen Mary University of London, London, UK

The results show very good RIN figures by careful attention to detail to inhibit skin RNAases and prevent degradation of RNA etc. The transcriptomic profiling of skin disease for research purposes is well established and it would improve the article to have clarity about the innovative features of this methodology compared to other published studies. The preparation of normal skin has also resulted in very good RIN here but there could be more detail about this. Has the biopsy been treated whole/ fat removed etc? Were the 10 30-micron curls used for normal skin also. Laser capture (discussed) is probably not a competitor for this in skin as heating results in lower RNA integrity. Cylindroma and trichoepithlioma are very cellular tumours not hyperkeratotic lesions: would this work in much commoner keratotic tumours? Such a method is of course mainly suitable for research studies and clinical trials not for routine pathology labs where transcriptomic profiling is needed on FFPE sections.

I have read this submission. I believe that I have an appropriate level of expertise to confirm that it is of an acceptable scientific standard.

# Peer review: Referee response for version 1

**DOI:** 10.21956/wellcomeopenres.15623.r32972

**Published:** 2018-04-24

**Authors:** Veronica A. Kinsler

**Affiliations:** 1 Pediatric Dermatology, Great Ormond Street Hospital for Children, London, UK; 2 Genetics and Genomic Medicine, UCL Institute of Child Health, London, UK

Overall this is an excellent contribution to technical knowledge of handling of skin samples, and a well-presented methods paper in so far as the optimised extraction method goes.

 I have a few points to be addressed:

1. You are not comparing extraction techniques here, but sample preparation. In this context, there is inadequate information on the handling of the "control" skin samples - detail is needed on how these were taken, how long they were left (and in what?) before extraction. If an RNA stabilisation solution was not used as a comparator this should be addressed, as really this would be the current gold standard for comparison, not direct extraction from a tissue sample that had been left in the fridge or in culture medium. In addition it is not clear whether the normal skin biopsies were also 4-6mm, or whether these were all 4mm.
2. It is possible to divide even a 4mm skin biopsy for histology and RNA extraction and still get enough for a library prep, so the claim that this method is the only one which would allow histology is not correct
3. It is possible that tumours and normal skin differ intrinsically in the amount of RNA produced, and that this is responsible for the differences in quantity, particularly as the quality of the RNA is the same from tumour and normal skin. It would have been much better to use half of each tumour biopsy for each preparation method, and if this could be done on 10 samples going forward you would have a much stronger paper. If not possible in the clinical context this point at least needs to be addressed specifically in the discussion.

I have read this submission. I believe that I have an appropriate level of expertise to confirm that it is of an acceptable scientific standard, however I have significant reservations, as outlined above.
